# Neck/shoulder discomfort due to visually demanding experimental near work is influenced by previous neck pain, task duration, astigmatism, internal eye discomfort and accommodation

**DOI:** 10.1371/journal.pone.0182439

**Published:** 2017-08-23

**Authors:** Camilla Zetterberg, Mikael Forsman, Hans O. Richter

**Affiliations:** 1 Centre for Musculoskeletal Research, Department of Occupational and Public Health Sciences, Faculty of Health and Occupational Studies, University of Gävle, Gävle, Sweden; 2 Institute of Environmental Medicine, Karolinska Institutet, Stockholm, Sweden; Universitat Politecnica de Catalunya, SPAIN

## Abstract

Visually demanding near work can cause eye discomfort, and eye and neck/shoulder discomfort during, e.g., computer work are associated. To investigate direct effects of experimental near work on eye and neck/shoulder discomfort, 33 individuals with chronic neck pain and 33 healthy control subjects performed a visual task four times using four different trial lenses (referred to as four different viewing conditions), and they rated eye and neck/shoulder discomfort at baseline and after each task. Since symptoms of eye discomfort may differ depending on the underlying cause, two categories were used; *internal eye discomfort*, such as ache and strain, that may be caused by accommodative or vergence stress; and *external eye discomfort*, such as burning and smarting, that may be caused by dry-eye disorders. The cumulative performance time (reflected in the temporal order of the tasks), astigmatism, accommodation response and concurrent symptoms of internal eye discomfort all aggravated neck/shoulder discomfort, but there was no significant effect of external eye discomfort. There was also an interaction effect between the temporal order and internal eye discomfort: participants with a greater mean increase in internal eye discomfort also developed more neck/shoulder discomfort with time. Since moderate musculoskeletal symptoms are a risk factor for more severe symptoms, it is important to ensure a good visual environment in occupations involving visually demanding near work.

## Introduction

The steadily increasing use of computers, tablets and smartphones, both for work and leisure, has led to health problems, of which the most frequently reported by professional users concerns the eyes (i.e., asthenopia), including discomfort, strain, fatigue, tired, burning, red and/or irritated eyes, and blurred and double vision [1,2], in addition to neck/shoulder discomfort [3–5]. Such symptoms involving the eyes and/or neck/shoulder discomfort are influenced by age, gender and previous occurrence [6–9]. Eye-related symptoms specifically are affected by visual demands and dry eyes [1,2,10], while neck/shoulder discomfort arises from, e.g., awkward (non-neutral) postures, prolonged sitting in front of the computer, repetitive tasks, and static muscle activity [6,7,11].

Although these two types of symptoms are often studied separately, there are indications that they can be related physiologically [1,3,12]. For example Wiholm and colleagues (2007) found that eye- and neck/shoulder discomfort coexisted in 21% of their subjects [13]. A survey of telecom workers revealed that ocular symptoms, wearing spectacles and vergence disparity were associated with a higher frequency of neck/shoulder problems [8]. An intervention study demonstrated that postmen experiencing eye strain reported more shoulder discomfort than those without eye strain, and that appropriate visual ergonomics reduced both eye and musculoskeletal discomfort in both groups [14,15].

In an experimental investigation, uncorrected astigmatism was found to aggravate eye discomfort associated with reading from a computer screen [16], and in the clinic convergence insufficiency is considered to be a cause of muscular discomfort, cf. [17]. It has also been proposed that muscular activity in the neck and shoulders is increased by high visual demands. Thus, muscular activity in the neck and shoulders of participants suffering from work-related upper extremity disorders was attenuated when the visual demands were lowered by replacing habitual with appropriate optical corrections [18]. Moreover, a number of experiments have showed that the level of eye-lens accommodation affects muscular activity in the neck and shoulders [18–20].

Most research on the association between eye and neck/shoulder discomfort has been cross-sectional or interventional. In the former it is difficult to control relevant factors, and in the latter several changes are often introduced simultaneously, e.g., improved lighting and corrective eyewear or ergonomic training and adjustment of the workstation [12,15]. Such designs make it difficult to determine the impact of individual factors, cf. [21,22].

Therefore, we set out to evaluate the impact of visually demanding experimental near work on the immediate onset of neck/shoulder discomfort, while controlling for or excluding factors known to be relevant, such as awkward postures and repetitive tasks (e.g., working with a keyboard and/or mouse). To this end participants with and without chronic neck pain performed a standardised visual task at a computer screen under four different viewing conditions using four different trial lenses, and rated their eye and neck/shoulder discomfort at baseline, and after each task. Our specific research questions were: Does eye and/or neck/shoulder discomfort increase during visually demanding experimental near work? If so, does the viewing condition or other factors exert an impact? And are there any differences between individuals with and without chronic neck pain in these respects?

## Methods

Data for this study were collected as part of a larger project. For further details on the overall methods, please see Zetterberg et al. 2013 [20].

### Participants

Thirty-three participants suffering from chronic neck pain (the *neck group*: 27 women and 6 men, median age 39 years, range 20–47) and 33 healthy age- and gender matched controls (the *control group*: 27 women and 6 men, median age 37, range 19–47) were included in this study. Prior to participation all individuals but three (one in the control group and two in the neck group) were examined by a licensed optometrist. The examination included for example assessment of refractive errors and visual acuity. All participants were provided with verbal and written descriptions of the experimental protocol and gave their written informed consent to participate. This study was pre-approved by the Medical Ethical Review Board of Uppsala University (2006:027) and conducted in accordance with the Declaration of Helsinki.

### Procedure

The participants visited the laboratory on a single occasion, and they performed visually demanding experimental near work on a computer screen. The session began with preparations involving measurement of refractive errors (Power Refractor R03, Plusoptix, Nürnberg, Germany) [23] and selection of lenses for the various viewing conditions. Any spherical refractive errors (i.e., myopia or hyperopia) detected were corrected for (in steps of 0.25 D) by the trial lenses. Cylindrical refractive errors (i.e., astigmatism) were not corrected for during the experiment. In addition, surface electrodes for electrocardiography (ECG) were placed laterally to the left and right of the sixth rib and other electrodes for electromyography (EMG) bilaterally on the descending portion of the upper trapezius muscle followed by performance of three submaximal normalisation contractions [24], see Zetterberg et al. 2013 for details [20].

Thereafter, the participant was seated in an office chair in front of the computer screen and his/her posture adjusted for comfort, with support for the head and back. The upper arms hung alongside the trunk, with the hands resting on the lap. To obtain a large amount of information from the auto refractor, he/she was instructed to sit relaxed, maintain the same posture and minimise movement.

**Fig 1:**
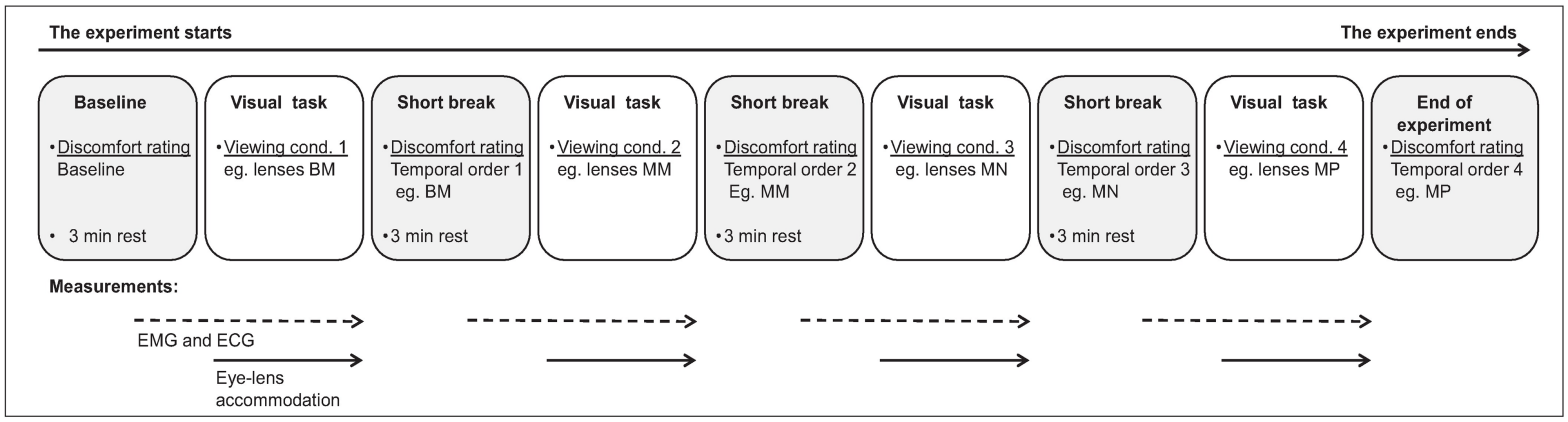
The experimental design. A standardised visual task was repeated four times with different trial lenses (BM = binocular minus, MM = monocular minus, MN = monocular neutral and MP = monocular plus). Lens order was randomized using a Latin square. A 3-min rest period preceded each visual task. Internal and external eye discomfort, and neck/shoulder discomfort were rated on Borg’s CR-10 scale at baseline and after each visual task. EMG and ECG were measured during rests and visual tasks, and eye-lens accommodation was measured during visual tasks.

A 7-minute standardised visual task was repeated four times with four different trial lenses (viewing conditions). A 3-minute rest period preceded each session during which the participant sat relaxed and leaned back with eyes closed. ECG, EMG and eye-lens accommodation (measured with the auto refractor) were recorded. Before the experiment began (at baseline) and immediately after each viewing condition, the participants rated their eye and neck/shoulder discomfort on Borg’s CR-10 scale [25,26]. The experimental design is illustrated in Fig 1.

### The visual task and the viewing conditions

**Fig 2:**
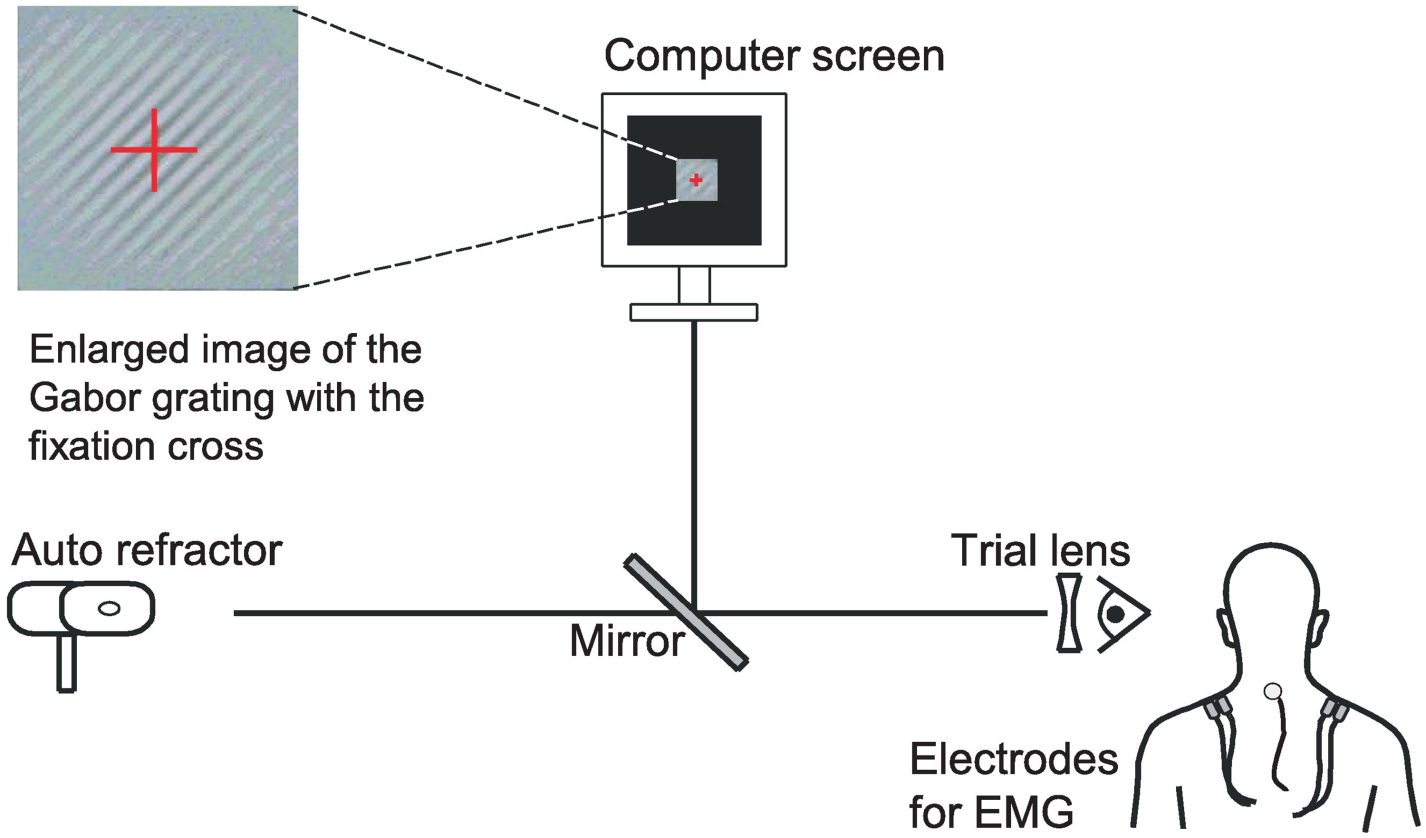
Laboratory set-up. **S**chematic illustration of the experimental set-up in the laboratory.

The standardised visual task required sustained fixation on a black-and-white Gabor grating of varying contrast displayed on a computer screen (Sony F520 CRT monitor and a VSG video board, Cambridge Research System Ltd, Rochester, UK) [27]. The size of the grating was 6 x 6 cm, the distance to this screen was 65 cm (1.5 diopters) and the gaze angle approximately 15° downwards. At the start of this task, the grating contrast was zero and this contrast began to increase. As soon as the participant perceived the grating, he or she pushed a hand-held button to freeze contrast for a short period, after which the contrast started to decrease again until the participant pushed the button one more time to indicate that the granting had become invisible. This procedure was repeated for 7 minutes. A schematic model of the experimental set-up in the laboratory is depicted in Fig 2.

Four viewing conditions were utilised to create different types of visual demands using four different trial lenses: Monocular Neutral (MN) wearing 0 D trial lenses; Binocular Minus (BM) and Monocular Minus (MM) wearing -3.5 D trial lenses to facilitate increased accommodation; Monocular Plus wearing +3.5 D trial lenses to facilitate decreased accommodation. During the monocular conditions, the non-dominant eye was occluded with a black lens. The order in which these viewing conditions occurred was randomised among the participants using a Latin square. In total, each participant performed 28 minutes (4 x 7 minutes) of visually demanding near work.

During the experiment the room was dark and the only light came from the 6 x 6 cm Gabor grating. The rest of the screen was covered with black cardboard paper.

### Data recording and processing

#### Eye-lens accommodation

Eye-lens accommodation was quantified during the visual tasks with the auto refractor (sampling frequency 25 Hz) [28,29], allowing calculation of an individual *accommodation response* (mean, in diopters, D) for each viewing condition.

#### Eye and neck/shoulder discomfort

Before the experiment began (baseline) and after each viewing condition each participant rated his/her eye and neck/shoulder discomfort on Borg’s CR-10 scale (Table 1) [25,26]. The literature contains more than 200 ways of describing symptoms related to eye discomfort [30] and it has been suggested that symptoms or descriptors of eye discomfort differ depending on the underlying cause. It is suggested that symptoms like ache or strain may be caused by accommodative or vergence stress (referred to as internal symptoms), and that symptoms like burning or smarting may be caused by dry-eye disorders (referred to as external symptoms [31]. Therefore, two questions were rated and the question for internal symptoms was “*To what extent do your eyes ache or feel strained*?*”* and the question for external symptoms was *“To what extent do you have a burning or smarting sensation in your eyes*?*”*. In the case of neck/shoulder discomfort one question was rated: *“To what extent do you feel pain or discomfort in your neck and/or shoulders*?”

**Table 1:** Borg’s CR-10 scale along with the written descriptors.

Borg’s CR-10 scale	
0	Nothing at all
0.5	Extremely weak
1	Very weak
2	Weak
3	Moderate
4	
5	Strong
6	
7	Very strong
8	
9	
10	Extremely strong
•	Maximal

The individual differences in ratings of eye discomfort, for both internal (Int) and external (Ext) symptoms, from before the experiment (baseline) to after the fourth viewing condition (ΔIntEye and ΔExtEye) and to after the various viewing conditions (ΔIntEye_MN_, ΔIntEye_BM_, ΔIntEye_MM_, ΔIntEye_MP_ and ΔExtEye_MN_, ΔExtEye_BM_, ΔExtEye_MM_, ΔExtEye_MP_) were calculated. The same were calculated for neck/shoulder discomfort (ΔNeck and ΔNeck_MN_, ΔNeck_BM_, ΔNeck_MM_, ΔNeck_MP_).

#### Electrocardiography and electromyography

ECG and EMG were recorded both during rests and visual tasks, the signals being amplified, band-pass filtered (ECG: 0.05–35 Hz, EMG: 10–500 Hz) and sampled at 2000 Hz (EMG100C, BIOPAC Systems, Inc., Santa Barbara, CA, USA). ECG was used to assess the heart rate variability (HRV) as an indicator of autonomic reactivity (e.g., due to stress) during the experiments. The variation in the length of intervals between consecutive heartbeats can be utilised to quantify autonomic heart regulation, as well as balance between sympathetic and parasympathetic activation [32]. The standard deviation in the length of the periods between R peaks (SDNN) was used here to quantify HRV. The individual means in HRV for the various viewing conditions were calculated for statistical analysis.

The raw EMG signals were first filtered to eliminate disturbance from heart signals [20,33], then converted by the root-mean-square (RMS) procedure, normalised to submaximal reference contractions, and expressed as %RVE (reference voluntary electrical activity) [24]. The 10^th^ percentile of the normalised RMS-values was utilised as an indicator of static muscular activity [19,34,35]. For statistical analyses, the individual differences (in %RVE) between rest and the various viewing conditions were calculated.

### Data analyses

All statistical analyses were performed with the PASW 20.0 program for Windows (SPSS Inc., Chicago, IL, USA). All data sets were tested for normality with the Kolmogorov-Smirnov test and statistical tests chosen on the basis of the distribution. The level of significance was set at α = 0.05.

The Wilcoxon sign rank test was employed to explore whether there was a difference in the rating of eye and neck/shoulder discomfort from baseline to after the fourth viewing condition within the groups (control and neck), and the groups compared in this respect with the Mann Whitney U-test (using ΔIntEye, ΔExtEye and ΔNeck). The Friedman test was used to identify differences in eye and neck/shoulder discomfort within each group under the various viewing conditions (using ΔIntEye_MN_, ΔIntEye_BM_, ΔIntEye_MM_, ΔIntEye_MP_, ΔExtEye_MN_, ΔExtEye_BM_, ΔExtEye_MM_, ΔExtEye_MP_ and ΔNeck_MN_, ΔNeck_BM_, ΔNeck_MM_, ΔNeck_MP_), and the Wilcoxon signed rank test for post hoc examination. Mann Whitney U-test was utilised to analyse corresponding differences between the two groups.

Since auto-correlation in time series can be assumed (i.e., neighbouring values are likely to be correlated) and since some of our variables were not distributed normally, the General Estimating Equation (GEE) for repeated measurements was applied to search for factors that contribute to the variation in neck/shoulder discomfort during visually demanding experimental near work [36–39]. For this purpose, the participants were classified according to the spherical and cylindrical refractive errors determined by the optometrist (spherical: *emmetrope* 0 D (± 0.25), *myope* ≥ -0.5 D, *hyperope* ≤ 0.5 D; and cylindrical: *astigmatic* ≥ -0.5 D, *non-astigmatic* < -0.5 D). Three participants were not examined by the licensed optometrist. Those three were classified for spherical refractive errors on the basis of the measurement with the auto refractor, and considered missing with the respect to the cylindrical refractive error. For one participant no data on cylindrical error was available. To reduce the number of missing data concerning accommodation response, three categories were formed for each viewing condition reflecting the extent of accommodation: *low accommodation*, below the mean; *high accommodation*: above the mean; and *missing accommodation* where the number of data points from the auto refractor was insufficient (< 25% of the total data).

The dependent variable in the GEE was the difference in *neck/shoulder discomfort* from baseline to the various viewing conditions (ΔNeck_MN_, ΔNeck_BM_, ΔNeck_MM_, ΔNeck_MP_). The independent parameters were *group* (control or neck), *temporal order* of the ratings of discomfort after each viewing condition (temporal order 1, 2, 3 or 4), *accommodation response* (low, high or missing), *refractive error* (emmetrope, myope or hyperope), and *cylindrical error* (astigmatic or non-astigmatic). The covariates were the difference in *internal and external eye discomfort* from baseline to after the various viewing conditions (ΔIntEye_MN_, ΔIntEye_BM_, ΔIntEye_MM_, ΔIntEye_MP_ and ΔExtEye_MN_, ΔExtEye_BM_, ΔExtEye_MM_, ΔExtEye_MP_), *heart rate variability* and *trapezius muscle activity*.

The GEE was performed two times to analyse the main effects of the variables. In model 1, all independent variables were analysed. In model 2, only variables making a significant contribution (p < 0.1) were included. Thereafter, interaction effects between the temporal order of the ratings of discomfort and the contributing variables from model 2 were explored in a third model (model 3) by adding one interaction term at a time to model 2. To check the assumption of normality, residuals from all these models were visualised as histograms and normal probability plots.

## Results

The refractive status of the participants is documented in Table 2. The mean accommodation responses and heart rate variability under each viewing condition, and the mean difference in trapezius muscle activity between rest and the various viewing conditions are also presented in Table 2.

**Table 2:** Descriptive statistics. The refractive errors of our participants, accommodation responses (D) and heart rate variability during the various viewing conditions, and the mean difference in trapezius muscle activity between rest and the various viewing conditions. The difference between the groups were analysed with Mann-Whitney U test. Unless otherwise indicated, the values presented are means with standard deviation in brackets (n = number of participants for whom reliable information was available).

Parameter	Viewing condition	Control group	n	Neck group	n	p-value
Spherical error (n) (emmetrope/myope/hyperope)	-	14/12/7	33	16/13/4	33	0.49
Cylindrical error (n) (non-astigmatic/astigmatic)	-	19/13	32	13/17	30	0.21
Accommodation response	MN	1.49 (0.68)	31	1.51 (0.74)	25	0.90
	BM	2.75 (1.90)	23	3.39 (2.09)	20	0.53
	MM	3.15 (1.64)	25	3.68 (2.00)	23	0.53
	MP	0.98 (0.84)	29	0.97 (0.99)	27	0.72
Heart rate variability	MN	42.7 (13.9)	33	41.4 (29.5)	33	0.20
	BM	44.4 (14.8)	33	41.2 (29.7)	33	0.07
	MM	43.7 (13.6)	33	39.4 (28.1)	33	0.03
	MP	42.5 (13.8)	33	40.9 (29.3)	33	0.11
Trapezius muscle activity	MN	0.27 (0.79)	31	0.12 (0.96)	32	0.13
	BM	0.17 (0.64)	31	0.21 (0.43)	32	0.29
	MM	0.26 (0.55)	30	0.24 (1.37)	32	0.11
	MP	0.53 (0.89)	30	0.00 (0.59)	31	0.02

MN = monocular neutral, BM = binocular minus, MM = monocular minus, MP = monocular plus.

**Fig 3:**
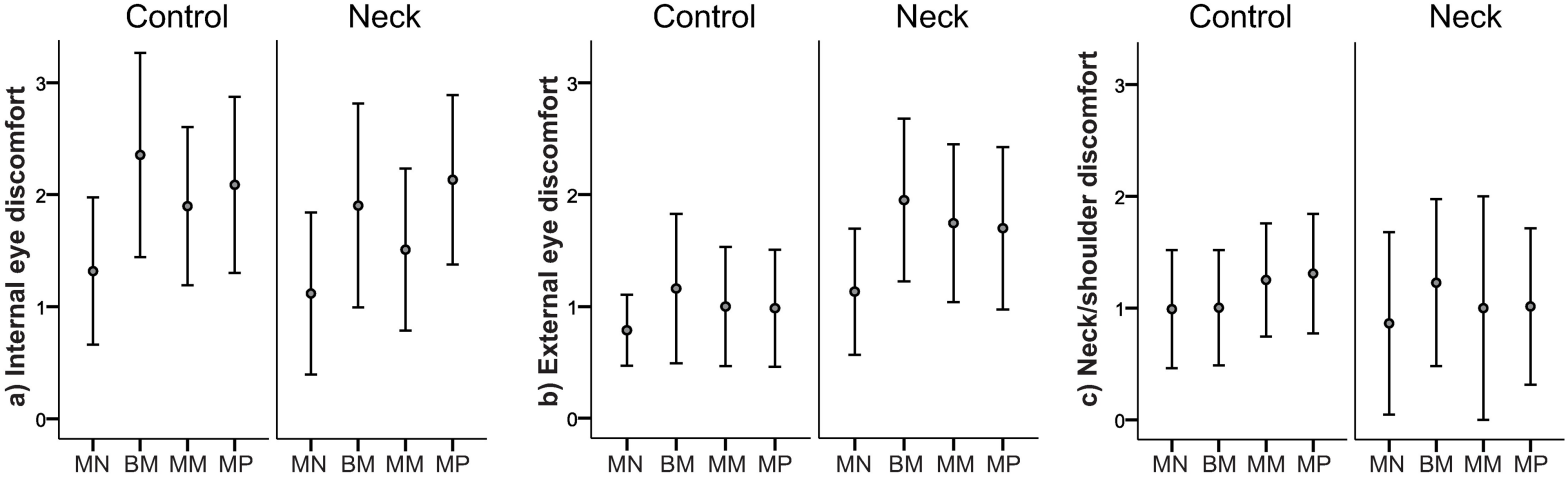
Difference in discomfort between baseline and the viewing conditions. The differences in the ratings of a) internal eye discomfort b) external eye discomfor, and c) neck/shoulder discomfort by the control and neck groups from baseline to after the various viewing conditions (ΔIntEye_MN_, ΔIntEye_BM_, ΔIntEye_MM_, ΔIntEye_MP_, ΔExtEye_MN_, ΔExtEye_BM_, ΔExtEye_MM_, ΔExtEye_MP_, and ΔNeck_MN_, ΔNeck_BM_, ΔNeck_MM_, ΔNeck_MP_). The values presented are means with 95% confidence intervals. MN = monocular neutral, BM = binocular minus, MM = monocular minus, MP = monocular plus.

Self-reported internal and external eye discomfort and neck/shoulder discomfort at baseline, and following the four viewing conditions are shown in Table 3. Since the order of the viewing conditions (i.e. the order of the trial lenses) was randomised, the ratings of discomfort are presented in two ways; first as the temporal order of the rating (1, 2, 3 and 4), and then as the rating after a specific viewing condition (MN, BM, MM, MP). The differences in the discomfort ratings from baseline to after the fourth viewing condition (ΔEyeInt, ΔEyeExt and ΔNeck) are shown in Table 4. The differences in ratings of internal and external eye discomfort and neck/shoulder discomfort from baseline to after the specific viewing conditions are depicted in Fig 3.

**Table 3:** Eye and neck/shoulder discomfort. The ratings of internal and external eye discomfort and and neck/shoulder discomfort by the control and neck groups at baseline following the four viewing conditions presented as the temporal order of the rating (1, 2, 3 and 4), and as the rating after a specific viewing condition (MN, BM, MM, MP). The values presented are medians with the range in brackets.

Temporal	Internal eye discomfort^1^		External eye discomfort^2^		Neck/shoulder discomfort	
order / VC	Controls	Neck group	Controls	Neck group	Controls	Neck group
Baseline	0.0 (0–2.0)	0.3 (0–4.0)	0.0 (0–2.0)	0.3 (0–7.0)	0.0 (0–2.0)	2.5 (0.5–9.0)
1	0.5 (0–6.0)	2.0 (0–7.0)	0.3 (0–4.0)	2.0 (0–7.0)	0.5 (0–5.0)	2.0 (0.5–8.0)
2	1.0 (0–3.0)	3.0 (0.5–9.0)	1.0 (0–5.0)	2.5 (0–7.0)	1.0 (0–4.0)	3.0 (0.5–9.0)
3	1.5 (0–6.0)	2.5 (0.5–9.0)	1.0 (0–7.0)	2.5 (0–7.0)	2.0 (0–7.0)	4.0 (1.0–9.0)
4	2.0 (0–7.0)	3.0 (0.5–7.0)	1.5 (0–5.0)	2.5 (0–7.0)	2.0 (0–5.0)	5.0 (2.0–10.0)
MN	1.5 (0–7.0)	2.0 (0–7.0)	1.0 (0–3.0)	2.0 (0–7.0)	1.5 (0–7.0)	3.0 (1.0–10.0)
BM	2.0 (0–7.0)	3.0 (0–9.0)	0.5 (0–7.0)	3.0 (0–7.0)	1.0 (0–6.0)	3.0 (1.0–9.0)
MM	2.0 (0–6.0)	2.0 (0–6.0)	1.0 (0–5.0)	2.5 (0.3–7.0)	1.5 (0–5.0)	3.0 (0.5–10.0)
MP	2.0 (0–9.0)	3.0 (0–9.0)	1.0 (0–5.0)	2.5 (0–7.0)	2.0 (0–5.0)	3.0 (0.5–9.0)

^1^ To what extent do your eyes ache or feel strained?

^2^ To what extent do you have a burning or smarting sensation in your eyes?

VC = viewing condition,

MN = monocular neutral, BM = binocular minus, MM = monocular minus, MP = monocular plus.

**Table 4:** Difference in discomfort ratings. The differences in the discomfort ratings from baseline to after the fourth viewing condition (temporal order 4) for internal and external eye discomfort and for neck/shoulder discomfort (ΔIntEye, ΔExtEye and ΔNeck). The difference between the groups were analysed with Mann-Whitney U test.

	Control group		Neck group		
	Median	Range	Median	Range	p-value
Internal eye discomfort^1^	1.0	0.3–2.2	2.5	1.0–4.0	0.02
External eye discomfort^2^	1.0	0.0–2.0	1.7	0.2–2.7	0.20
Neck/shoulder discomfort	1.5	0.5–2.7	2.0	0.5–4.0	0.26

^1^ To what extent do your eyes ache or feel strained?

^2^ To what extent do you have a burning or smarting sensation in your eyes?

The Wilcoxon sign rank test revealed an increase in the ratings of both internal and external eye discomfort and neck/shoulder discomfort from baseline to after the fourth viewing condition (temporal order 4) within both groups (ΔIntEye, ΔExtEye, and ΔNeck p < 0.001). The Mann Whitney U-test revealed differences between groups for internal eye discomfort, but not for external discomfort or neck/shoulder discomfort (see Table 4).

The Friedman test revealed differences in the ratings of internal eye discomfort between the specific viewing conditions (ΔIntEye_MN_, ΔIntEye_BM_, ΔIntEye_MM_, ΔIntEye_MP_) for both groups (controls p = 0.011, neck group p = 0.003). There were no significant differences in external eye discomfort between the specific viewing conditions in any of the groups (p ≥ 0.098). Post hoc examination with the Wilcoxon signed rank test showed a more pronounced rise in the ratings of internal eye discomfort from baseline to after the viewing condition with the three more demanding lenses (BM, MM, MP) for the control group (p ≤ 0.019)), than with the less demanding neutral lens (MN) (Fig 3A, left panel). For the neck group there were significant differences for two of the more demanding lenses (BM and MP, p ≤ 0.003) compared with the neutral lens (Fig 3A, right panel). There were no significant differences in the ratings of neck/shoulder discomfort for the various viewing conditions by either group (p > 0.3) (Fig 3C).

The Mann Whitney U-test identified differences between the two groups with respect to the differences in their ratings of external eye discomfort (smarting and burning) from baseline to after the specific viewing conditions with two of the more demanding lenses (ΔExtEye_BM_ p = 0.044, and ΔExtEye_MP_ p = 0.016). In contrast, the differences in the rating of internal eye discomfort (ache and strain) and neck/shoulder discomfort from baseline to after the specific viewing condition were similar for the two groups (p > 0.4).

**Fig 4:**
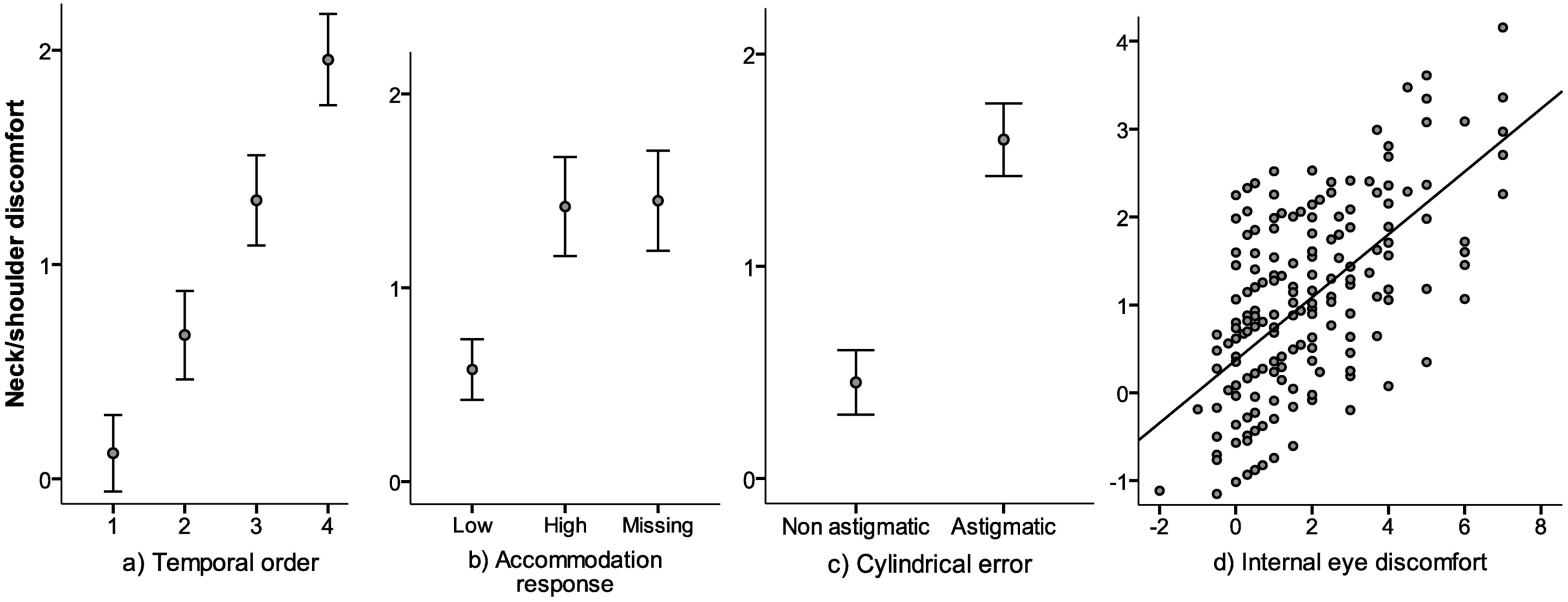
GEE model 2. The relationships between the mean value of the dependent variable (the difference in the ratings of neck/shoulder discomfort from baseline to after the various viewing conditions: ΔNeck_MN_, ΔNeck_BM_, ΔNeck_MM_, ΔNeck_MP_) predicted from the GEE analysis (model 2) and a) temporal order of the ratings of discomfort, b) accommodation response, c) the cylindrical error, and d) the corresponding difference the ratings of internal eye discomfort.

**Fig 5:**
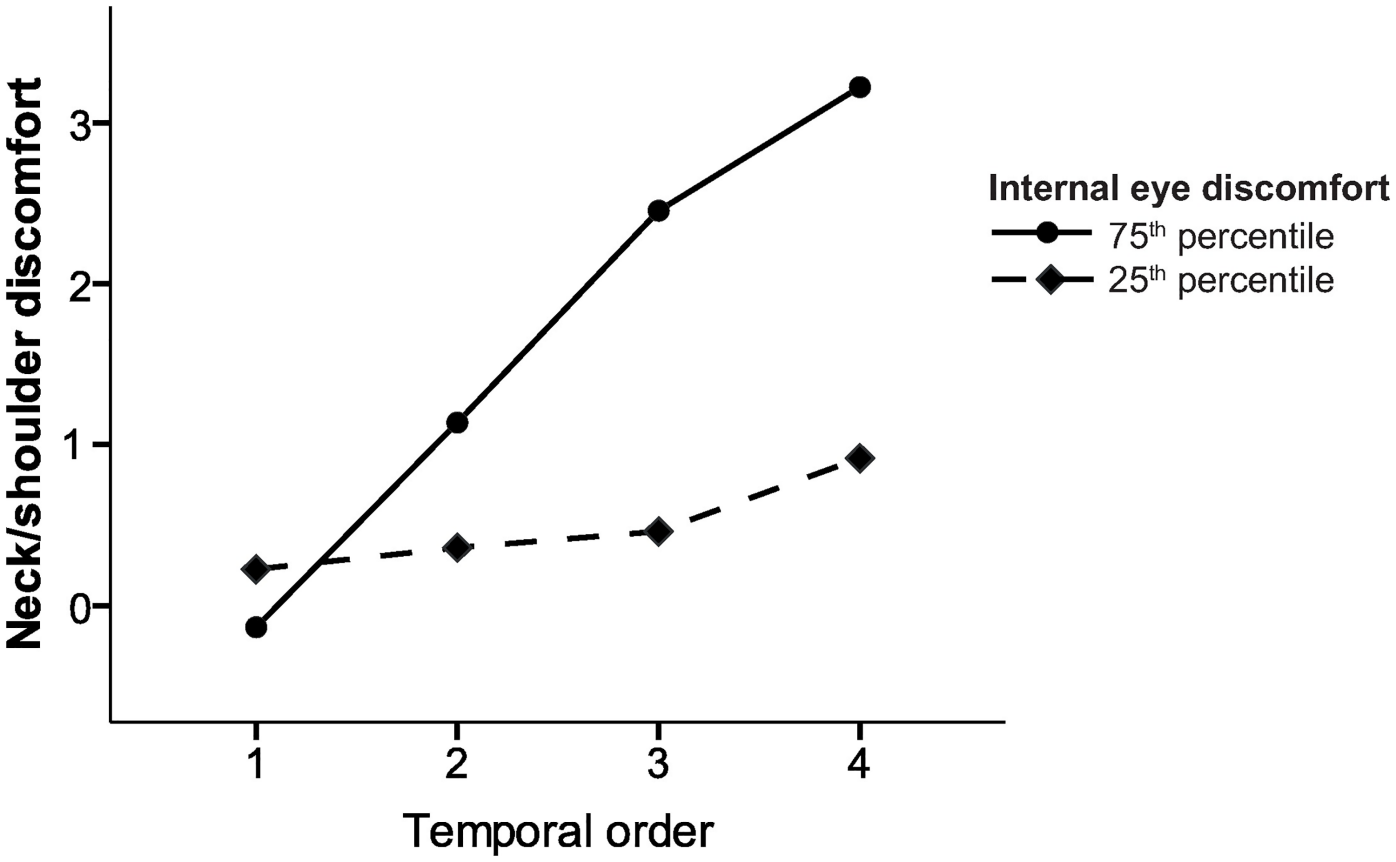
Interaction effect from GEE model 3. The interaction effect of the temporal order of the viewing conditions and the difference in the ratings of internal eye discomfort from baseline to the various viewing conditions on the mean predicted rating of neck/shoulder discomfort (GEE model 3). To illustrate the difference between small and large differences in internal eye discomfort the participants were divided into the 25^th^ percentile (dotted line, mean difference -0.04, n = 17), and the 75^th^ percentile (solid line, mean difference4.33, n = 17) using the variable ΔIntEye.

The GEE analyses are presented in Tables 5 and 6 and Figs 4 and 5. Model 1 revealed that the temporal order of the ratings of discomfort, concurrent self-reported internal eye discomfort, astigmatism, and the extent of accommodation all exerted an impact (p < 0.1) on the variance in neck/shoulder discomfort during the visually demanding near work. The variables group, spherical refractive errors, external eye discomfort, heart rate variability, and trapezius muscle activity had no influence and were excluded from the second model, which confirmed the main effects of the variables included (p ≤ 0.023) (see Table 5 and Fig 4). In model 3 interaction effects between the temporal order of the ratings of discomfort, on the one hand, and the extent of accommodation, cylindrical error and internal eye discomfort, on the other, were explored by adding one interaction term at a time to model 2. The only significant interaction was *temporal order x internal eye discomfort* (see Table 6 and Fig 5). This interaction diminished the significant main effect of the temporal order (Tables 5 and 6).

**Table 5:** The GEE analyses in model 1–3. The dependent variable was the difference in the rating of neck/shoulder discomfort from baseline to after the four specific viewing conditions (ΔNeck_MN_, ΔNeck_BM_, ΔNeck_MM_, ΔNeck_MP_). In model 1 the main effects of all independent variables were analysed. In model 2, only the variables indicated by model 1 as making a significant contribution (p < 0.1) were included. Model 3 included the variables from model 2 and the interaction effect between temporal order and internal eye discomfort.

		Model 1 651*	Model 2 650*	Model 3 623*
Independent variable	df	p-value	p-value	p-value
Intercept	1	0.147	0.003	0.005
Group^a)^	1	0.262	-	-
Temporal order^a)^	3	< 0.001	< 0.001	0.108
Accommodation response^a)^	2	0.016	0.023	0.038
Spherical error^a)^	2	0.876	-	-
Cylindrical error^a)^	1	0.013	0.002	0.003
Internal eye discomfort^b^^,^ ^c)^	1	< 0.001	< 0.001	< 0.001
External eye discomfort^b^^,^ ^d)^		0.446		
Hart rate variability^b)^	1	0.761	-	-
Trapezius muscle activity^b)^	1	0.365	-	-
*Temporal order x Internal eye discomfort*	3	-	-	< 0.001

* = Goodness of fit, df = degrees of freedom

^a)^ Categorical variable,

^b)^ Continuous variable

^*c*)^ *To what extent do your eyes ache or feel strained*?

^*d*)^ *To what extent do you have a burning or smarting sensation in your eyes*?

**Table 6:** Estimated parameters by the GEE. Estimates of the parameters by the GEE including significant variables (model 2) and including interaction effects between temporal order and internal eye discomfort (model 3).

Parameter			Model 2		Model 3	
		n	Slope (β)	CI	Slope (β)	CI
Temporal order ^a)^	1 ^c)^	62	-	-	-	-
	2	62	0.45	0.09–0.80	0.24	-0.28–0.76
	3	62	1.10	0.65–1.55	0.54	0.02–1.06
	4	62	1.63	1.01–2.25	0.71	0.06–1.36
Accommodation response ^a)^	Low ^c)^	123	-	-	-	-
	High	68	0.65	0.13–1.17	0.58	0.11–1.06
	Missing	57	0.39	-0.15–0.65	0.41	-0.09–0.90
Cylindrical error ^a)^	Non astigmatic ^c)^	120	-	-	-	-
	Astigmatic	128	0.91	0.30–1.52	1.11	0.46–1.76
Internal eye discomfort ^b)^		248	0.27	0.14–0.41	0.06	-0.20–0.21
*Temporal order 1 x Internal eye discomfort* ^c)^					-	-
*Temporal order 2 x Internal eye discomfort*					0.16	-0.14–0.46
*Temporal order 3 x Internal eye discomfort*					0.39	0.13–0.65
*Temporal order 4 x Internal eye discomfort*					0.53	0.23–0.83

n = number of cases, CI = confidence interval

^a)^ Categorical variable,

^b)^ Continuous variable,

^c)^ Reference category

## Discussion

In this laboratory investigation visually demanding experimental near work increased both the internal and external eye discomfort, and the neck/shoulder discomfort, experienced by subjects with and without chronic neck pain. For the neck group the total increase in eye discomfort was higher than the increase experienced by the control group (see Table 4), but it was only significant for the internal symptoms (i.e. ache and strain) which may be caused by accommodative or vergence stress [31].

Differences in eye discomfort under the different viewing conditions were reported by both groups. This was also only evident for the internal symptoms. For the control group, the increase in internal eye discomfort from baseline to after the conditions with the three more demanding lenses (i.e., the minus lenses BM and MM and the plus lens MP) was greater than with the least demanding lens (the neutral lens MN) (Fig 3). For the neck group, the increase was greater for two of the more demanding lenses (BM and MP). Group differences were evident only for the external eye discomfort; the neck group reported higher increase from baseline to after the viewing conditions BM and MP (Fig 3). Regarding neck/shoulder discomfort there were no differences, neither among the various viewing conditions nor between groups.

The factor that explained most of the variation in neck/shoulder discomfort was the temporal order, i.e., the ratings of neck/shoulder discomfort increased from condition one to condition two, to three to four. Also significant were high accommodation during the visually demanding near work, astigmatism, and internal eye discomfort, whereas external eye discomfort, the group (control or neck), spherical error, heart rate variability and trapezius muscle activity exerted no impact. An interaction effect of the temporal order of the ratings of discomfort and internal eye discomfort on neck/shoulder discomfort was identified.

### Internal and external eye discomfort

As mentioned in the introduction symptoms like ache or strain in the eyes may be caused by accommodative or vergence stress (internal symptoms), and symptoms like burning or smarting eyes may be caused by dry-eye disorders (external symptoms) [31]. Here, the visually demanding experimental near work elevated both internal and external eye discomfort within both groups. Interestingly, there were only significant differences among the viewing conditions for the internal eye symptoms, and it was only the internal symptoms that exerted an impact on neck/shoulder discomfort. These findings validate the visual task used, since the task was developed to induce predominantly accommodative stress.

### The effect of temporal order

When discussing musculoskeletal pain in connection with computer work, the duration of such work is often considered to be a potential risk factor [6,40–42]. However, it is often the time spent using the key board and/or mouse that is most crucial in this context. In the present study, the participants sat relaxed, using neither a keyboard nor mouse, being subjected to visual demands only. As described earlier [20], the attenuated ability to accommodate following the experiment confirms that the visual tasks were indeed demanding. Even though the participants were exposed to visual demands only, cumulative time spent performing the visually demanding near work (reflected in the temporal order of the four visual tasks) was the variable that influenced the increase in neck/shoulder discomfort to the greatest extent.

Eye discomfort during near work is also affected by the duration of the work. Thus, Portello and colleagues found a positive correlation between eye symptoms and the time spent working on a computer [10], and Toomingas and co-workers showed that the incidence of eye symptoms was related to both ‘hours doing computer work on a normal workday’, and ‘time at the computer without a break of minimum 10 minutes’ [9]. Here the visually demanding near work resulted in an increase in both internal and external symptoms of eye discomfort, but only internal symptoms exerted a statistical effect on neck/shoulder discomfort. There was also an interaction effect of the temporal order and internal eye discomfort on self-reported neck/shoulder discomfort. Fig 5 shows that the more pronounced the increase in internal eye discomfort from baseline to the fourth viewing condition, the greater the increase in neck/shoulder discomfort with time (from temporal order 1 to 4).

### The effect of cylindrical error and accommodation response

Previous research has demonstrated that both uncorrected and induced astigmatism aggravates visual symptoms [16,43,44]. In the present case astigmatism in the participants was not corrected for during the visually demanding experimental near work and the GEE models revealed that subjects with uncorrected astigmatism experienced more neck/shoulder discomfort (cf. Fig 4C).

One of our hypotheses was that the nature of the viewing condition would affect the rating of both eye and neck/shoulder discomfort. This was confirmed for both groups but only for internal eye discomfort: The participants in the control group rated less internal eye discomfort after using the least demanding lens (MN) than after the three the more demanding lenses (BM, MM, MP), for the participants in the neck group the same was evident for two of the more demanding lenses (BM, MP). Even though there were differences in accommodation between the various viewing conditions, the different viewing conditions might not reflect the actual load on the visual system, thus, the objectively determined extent of accommodation might provide a more accurate indication of visual load. Accordingly, the variable accommodation response, but not the viewing conditions were included in the GEE analyses. All three models (analysing main and interaction effects) indicated that the extent of accommodation contributed significantly to the variance in neck/shoulder discomfort.

### Posture and neck/shoulder discomfort

It is well known that the eyes lead the body and visual demands can result in postural changes [45]. Awkward working postures and sustained muscle activity can in turn, cause discomfort in the neck/shoulder area [7,46]. Although our participants were instructed to sit in a relaxed position, leaning slightly backward, with head and back support, head and/or neck posture was not evaluated objectively so that postural loads cannot be ruled out completely as a putative cause for some of the neck/shoulder discomfort. However, the auto refractor is disturbed by movements and when this instrument could not collect data accurately; the test supervisor instructed the participant to return to a relaxed and backward leaning position. In addition, the level of trapezius muscle activity during the experiment was low (cf. Table 2 in Zetterberg et al. 2013 [20]), indicating an absence of awkward postures. Furthermore, since muscle activity was non-significant in the GEE model, it appears unlikely that the elevated neck/shoulder discomfort originated from awkward postural demands and/or increased muscle activity.

### Limitations

The ratings of eye and neck/shoulder discomfort might have been influenced by the fact that when two different body areas are rated at the same time, the participant might rate both equally. To confirm our data, Friedman tests were applied to analyse differences in internal and external eye symptoms and neck/shoulder discomfort at baseline and after the four visual tasks for each group. There were significant differences between the three dimensions of discomfort ratings for baseline for both groups, and for ratings at temporal order 3 and 4 for the neck group.

In the GEE analyses accommodation response rather than the viewing condition was utilised as an indicator of visual load. It should be noted that with the minus lenses (BM and MM), the visual system compensated for dioptric blur (see Table 2), which most likely reflects the accommodative load. The plus lens (MP) was intended to attenuate accommodation and, therefore, the response in this case might not reflect the load in the same manner. The subjects could not compensate for the dioptric blur induced by the plus lens, resulting in a blurred image that was probably per se a load on the visual system. Under all three viewing conditions intended to place demands on the visual system (BM, MM, MP), the ratings of eye discomfort by the control group were higher, and this was also evident for the neck group for two of the demanding conditions (BM, MP). This might have been due, at least in part, to the blur in the case of the plus lens.

With multivariate statistical analyses, the number of observations limits the number of predictors that can be included in a model. To avoid bias, 10–15 observations per predictor has been proposed to be a reasonable number [47,48]. Since this study had 66 participants (observations), the number of predictors (independent variables) had to be restricted. Although age and gender exert an impact on both eye and neck/shoulder discomfort [6,9], gender was excluded from the GEE because there were too few male participants and age was not included since it is correlated to the accommodation response. The remaining nine variables in the first GEE were considered potentially valuable as predictors and included, even though the recommended number of predictors was thereby exceeded somewhat.

### Practical implications

The time we spend doing near work tasks (e.g., at a computer, tablet or smartphone) both during work and leisure has increased dramatically. The present laboratory investigation reveals that the cumulative time spent performing such work (reflected in the temporal order of the four visual tasks) influences both eye and neck/shoulder discomfort. An interaction effect was also observed between the temporal order and internal eye discomfort, i.e., participants who experience a more pronounced increase in internal eye discomfort (from baseline to after the fourth visual task) also develop more neck/shoulder discomfort with time.

Even though the current results are in line with earlier studies [8,13,14], it should be remembered that the laboratory set-up differed from typical working conditions. Nonetheless, moderate musculoskeletal symptoms are a risk factor for more severe symptoms [49], and, despite its limitations, the present study provides additional evidence for the importance of a good visual environment in preventing musculoskeletal disorders in occupations involving demanding near work. Furthermore, number of hours spent performing such work should, if possible, be limited with frequent breaks (cf. Toomingas et al. 2014 [9]).

Finally, special attention should be focused on persons already suffering from neck/shoulder symptoms, both in clinical practice and in research, since, in this study, such individuals were more prone to develop symptom of eye discomfort related to accommodative demands during visually demanding near work.

## Conclusion

The visually demanding experimental near work elevated both eye and neck/shoulder discomfort significantly. The cumulative time spent performing such work (reflected here in the temporal order of the four visual tasks), astigmatism, extent of accommodation and concurrent symptoms of internal eye discomfort, such as ache and strain, all exerted an impact on neck/shoulder discomfort. In addition, individuals with chronic neck pain were more prone to develop both internal and external eye discomfort during visually demanding near work. Moreover, participants rating a greater increase in internal eye discomfort also experienced more neck/shoulder discomfort as the duration of the near work became longer.

Accordingly, visual demands may cause eye discomfort, and visual demands and eye discomfort together might increase neck/shoulder discomfort as the duration of the visually demanding near work increases. Since moderate musculoskeletal symptoms are a risk factor for more severe symptoms, it is important to ensure a good visual working environment preventing musculoskeletal disorders in occupations involving visually demanding near work.

## Supporting information

S1 File Dataset. (XLSX)
